# Over-expression of Arabidopsis *AtCHR23* chromatin remodeling ATPase results in increased variability of growth and gene expression

**DOI:** 10.1186/1471-2229-14-76

**Published:** 2014-03-25

**Authors:** Adam Folta, Edouard I Severing, Julian Krauskopf, Henri van de Geest, Jan Verver, Jan-Peter Nap, Ludmila Mlynarova

**Affiliations:** 1 Laboratory of Molecular Biology, Plant Sciences Group, Wageningen University and Research Centre, Droevendaalsesteeg 1, Wageningen 6708 PB, The Netherlands; 2 Laboratory of Genetics, Plant Sciences Group, Wageningen University and Research Centre, Wageningen, The Netherlands; 3 Applied Bioinformatics, Bioscience, Plant Research International, Plant Sciences Group, Wageningen University and Research Centre, Wageningen, The Netherlands; 4 Present address: Department of Toxigenomics, Maastricht University, Maastricht, The Netherlands; 5 Expertise Centre ALIFE, Institute for Life Science & Technology, Hanze University of Applied Sciences, Groningen, The Netherlands

**Keywords:** Arabidopsis, Chromatin remodeling, Growth, Gene expression, Variability, Robustness

## Abstract

**Background:**

Plants are sessile organisms that deal with their -sometimes adverse- environment in well-regulated ways. Chromatin remodeling involving SWI/SNF2-type ATPases is thought to be an important epigenetic mechanism for the regulation of gene expression in different developmental programs and for integrating these programs with the response to environmental signals. In this study, we report on the role of chromatin remodeling in Arabidopsis with respect to the variability of growth and gene expression in relationship to environmental conditions.

**Results:**

Already modest (2-fold) over-expression of the *AtCHR23* ATPase gene in Arabidopsis results in overall reduced growth compared to the wild-type. Detailed analyses show that in the root, the reduction of growth is due to reduced cell elongation. The reduced-growth phenotype requires sufficient light and is magnified by applying deliberate abiotic (salt, osmotic) stress. In contrast, the knockout mutation of *AtCHR23* does not lead to such visible phenotypic effects. In addition, we show that over-expression of *AtCHR23* increases the variability of growth in populations of genetically identical plants. These data indicate that accurate and controlled expression of *AtCHR23* contributes to the stability or robustness of growth. Detailed RNAseq analyses demonstrate that upon *AtCHR23* over-expression also the variation of gene expression is increased in a subset of genes that associate with environmental stress. The larger variation of gene expression is confirmed in individual plants with the help of independent qRT-PCR analysis.

**Conclusions:**

Over-expression of *AtCHR23* gives Arabidopsis a phenotype that is markedly different from the growth arrest phenotype observed upon over-expression of *AtCHR12*, the paralog of *AtCHR23*, in response to abiotic stress. This demonstrates functional sub-specialization of highly similar ATPases in Arabidopsis. Over-expression of *AtCHR23* increases the variability of growth among genetically identical individuals in a way that is consistent with increased variability of expression of a distinct subset of genes that associate with environmental stress. We propose that ATCHR23-mediated chromatin remodeling is a potential component of a buffer system in plants that protects against environmentally-induced phenotypic and transcriptional variation.

## Background

Plants have evolved finely orchestrated mechanisms to regulate their growth in response to the environment as a programmed part of their sessile life style. These mechanisms help them to cope with the (possibly adverse) environment at any period of their existence. Notably developing seedlings are vulnerable to short-term adverse environments [1,2]. As a result, plants display substantial variability of growth, a phenomenon also known as growth plasticity [3]. Such plasticity allows plants to optimize their growth and development according to the prevailing environmental conditions, ensuring the best possible strategy to complete their life cycle and propagate. Growth plasticity is potentially important for agronomic use as it affects yield and quality in unfavorable environments. Plasticity for a trait as growth is largely organized at the molecular level in which epigenetic mechanisms play a critical role [3]. Chromatin remodeling is part of the epigenetic machinery, next to DNA methylation, histone modification and small RNA-based mechanisms [4], that is an integral part of overall plant development and is associated with plant responses to biotic [5] and abiotic stress [6].

We have shown previously that the SWI/SNF2-type ATPase encoded by *AtCHR12* is involved in the regulation of growth of *Arabidopsis thaliana* upon perceiving abiotic stress, such as drought or higher temperature [7]. Arabidopsis plants over-expressing *AtCHR12* showed growth arrest of normally active primary buds, as well as reduced growth of the primary stem when stressed. Without stress, they were indistinguishable from the wild-type. The growth arrest response depended on the severity of the stress applied. Another SWI/SNF2-type ATPase, *SPLAYED* (*SYD),* was shown to be required for resistance against the necrotrophic pathogen *Botrytis cinerea*[5]*,* whereas a knockout of the *AtDRD1* ATPase gene showed increased susceptibility to fungal pathogen *Plectosphaerella cucumerina*[8]. The SWI/SNF2-type ATPases are believed to mediate the complex interplay between chromatin remodeling and the enzymes involved in DNA and histone modification. This underlines the importance of ATP-dependent chromatin remodeling in responses of plants to environmental stress.

In addition, such chromatin modifications play a regulatory role during development [9] in establishing epigenetic states with expression patterns that are tightly regulated in time and space*.* In animals, such epigenetic states are determined early during the development, while in plants epigenetic mechanisms also operate after embryonic development [10]*.* Several chromatin remodeling ATPase genes have a role in plant development. The CHD3-subfamily ATPase *PICKLE* (*PKL*) selectively regulates a suite of genes during embryogenesis, seed germination and root development [11-13]. Recently, this gene was identified as negative regulator of photomorphogenesis [14]. Out of four genes of the SWI/SNF2-subfamily of Arabidopsis ATPases [15], *SYD* and *BRM* are involved in various, partially overlapping, developmental processes, such as root and floral development or seed maturation [16-18]. The other two members of this subfamily, *AtCHR12* and *AtCHR23,* have roles in embryo and endosperm development. A nearly lethal *atchr12*/*atchr23* double mutant containing weak allele displayed a variety of severe pleiotropic morphological defects, including poor maintenance of shoot and root meristems [19]. Such ATPase-mediated chromatin modification establishes a level of gene regulation that is likely to integrate developmental programs with the response to environmental signals.

It is thought that epigenetic modifications help to establish a buffer against environmental perturbations [20] that results in the phenotypic robustness of the organism. Both in *Drosophila*[21] and in yeast [22-24] the deletion of chromatin regulator genes markedly increased the variability of the phenotype studied, indicating that proper chromatin modification may counteract genetic, environmental and/or stochastic perturbations [25,26].

We here report on the marked impact of over-expression of the *AtCHR23* gene on the phenotype of Arabidopsis in terms of growth, reaction to adverse environments and genome-wide expression levels. *AtCHR23* is a paralog of *AtCHR12*[27] of which the effects of over-expression were presented earlier [7]. Over-expression of *AtCHR23* results in reduced growth compared to wild-type Arabidopsis, but phenotypic details between *AtCHR12* and *AtCHR23* over-expression are notably different, showing sub-specialization of these two paralogs. The effect of *AtCHR23* over-expression is notably quantitative both in terms of growth phenotype as in terms of gene expression. The over-expression of *AtCHR23* increases the variability of growth and expression variability of subsets of genes in populations of identical plants. It emphasizes the important role of chromatin modification in the control of gene expression in plants. Based on these results, we propose that accurate and controlled expression of *AtCHR23* is required for the stability or robustness of growth. We propose that ATCHR23-mediated chromatin remodeling could be part of a buffer system in plants that protects against environmentally-induced phenotypic and transcriptional variation [20].

## Results

### Construction Arabidopsis mutants with altered *AtCHR23* expression

To generate transgenic Arabidopsis lines over-expressing the *AtCHR23* gene a construct containing 35S CaMV promoter and genomic sequence of *AtCHR23* (including 5’-UTR) from the accession Columbia (Additional file 1: Figure S1) was used for transformation of wild-type Arabidopsis (Col-0). Two single-copy transgenic lines were identified and analyzed in detail: *AtCHR23*-4ov and *AtCHR23*-5ov. In addition, transgenic lines over-expressing cDNA copy of *AtCHR23* fused in-frame to the *GFP* gene under the 35S CaMV promoter in front (Additional file 1: Figure S1) were generated. Two separate single-copy transgenic lines were identified and analyzed: *G*_*AtCHR23*-1ov and *G*_*AtCHR23*-3ov. A third type of over-expressing transgenic line was generated by transformation with the cDNA copy of *AtCHR23* including 5’-UTR fused in frame to *GFP* driven by the native *AtCHR23*-promoter (Additional file 1: Figure S1). For comparison, two loss-of-function T-DNA insertion lines affecting *AtCHR23* expression were obtained from the Arabidopsis Stock Center. Both knockout lines showed no expression of full length *AtCHR23* transcript. The data presented in this paper are from SALK_057856 that in the remainder of this paper will be designated as *atchr23*. The other insertion line gave similar results (data not shown).

### Over-expression of *AtCHR23* reduces the growth of roots and increases phenotypic variation

The growth dynamics of seedlings of the knockout (*atchr23*) and over-expressing lines of *AtCHR23* was analyzed with the help of a root elongation assay using vertical agar plates described previously [7]. Stratified seeds of wild-type and mutant plants germinated at approximately the same time and frequency*.* The lengths of the primary root and hypocotyl, as well as other phenotypic characteristics, were measured repeatedly during development in different environmental conditions. To prevent possibly confounding influences of the environment experienced by the previous generation [28], all comparisons were made using seeds from parental plants (both for the wild-type and for the mutants) grown at the same time and in the same environment. Assays were based on at least 40 roots per condition, with at most 16 roots (8 mutant; 8 wild-type) per agar plate and five agar plates per assay.

**Figure 1:**
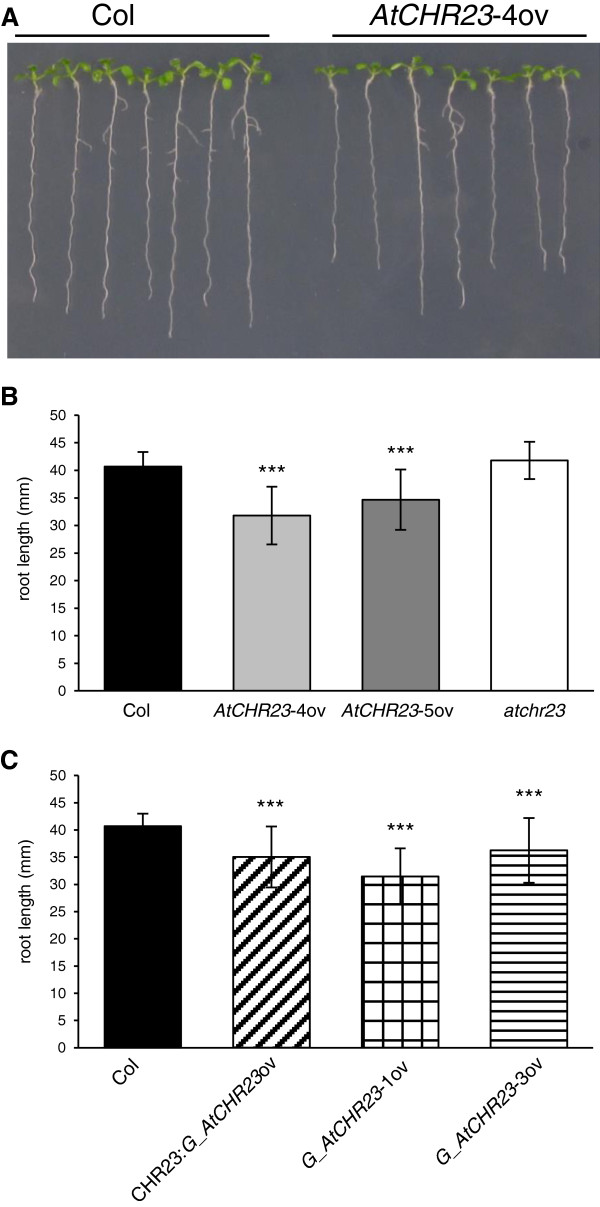
**Over-expression of *AtCHR23 *results in reduced root growth. (A)** Seedlings grown for eight days at 23°C, long-day (LD). **(B)** Mean (± SD) length of the primary root of Columbia wild-type (Col), knockout (*atchr23*) and two lines over-expressing the genomic copy of *AtCHR23*. **(C)** Mean (± SD) length of the primary root of Col wild-type and lines over-expressing the cDNA copy of *AtCHR23*. For each line, 40 seedlings were measured. Asterisks indicate significant differences from the wild-type: ^***^, P < 0.001.

Clearly visible differences between different lines were observed, notably with respect to the length of the root (Figure 1A). The differences in root length depended on the environmental conditions applied. When grown at 23°C under long-day conditions, roots of the two *AtCHR23*-ov mutants were considerably shorter than those of Columbia wild-type (Figure 1A and B). Data is summarized in Table 1. The average length of the roots of 8-day-old wild-type seedlings was 40.7 mm, whereas of *AtCHR23*-4ov seedlings it was 31.9 mm (21.6% reduction) and of *AtCHR23*-5ov 34.6 mm (14.9% reduction). Also up-regulation of *AtCHR23* with a cDNA copy of the gene (two *G*_*AtCHR23*-ov lines) resulted in seedlings with roots 14 and 22.7% shorter than wild-type, whereas the transgenic line with the native promoter showed 11% shorter roots (Figure 1C; Table 1). In such assays, the variation in the root length was considerable, with coefficients of variation (CV) ranging from 0.161 to 0.164 for over-expressing lines, whereas for wild-type it was 0.052 (Table 1). The variation of over-expressing mutants was significantly higher than in the wild-type (Levene’s test; Table 1). These data show that upon over-expression of *AtCHR23*, roots become not only significantly shorter, but also more variable and less uniform. In contrast, the knockout mutant *atchr23* develops roots that are only slightly longer than those of the wild-type (Figure 1B). In populations of 40 seedlings, this difference was not statistically significant. These root growth differences between the various *AtCHR23* mutants and the wild-type were consistently observed in several seed stocks that were produced in various growing conditions, greenhouse or growing chambers. Moreover, similar differences and variability patterns in root length were observed in seedlings grown at 18°C and 25°C (data not shown).

**Table 1:** **Root length reduction and *AtCHR23 *** **mRNA up-regulation in transgenic Arabidopsis lines with modified *AtCHR23 *** **expression**

**Plant line**	**Root length (mm)**^**a**^	**CV**^**b**^	**VAR**^**c**^	**P(VAR)**^**d**^	**Reduction in root length (%)**^**e**^	**Fold up-regulation *AtCHR23***^**f**^
Columbia – WT	40.53	0.052	4.76	na	na	na
*AtCHR23*-4ov	31.89	0.164	27.63	^***^	21.6	30
*AtCHR23*-5ov	34.65	0.161	31.32	^***^	14.9	40
*atchr23*	41.81	0.080	12.26	^*^	nd	na
*G_AtCHR23*-1ov	35.04	0.161	31.46	^***^	14.0	15
*G_AtCHR23*-3ov	31.46	0.163	26.49	^***^	22.7	13
CHR23:*G_AtCHR23*ov	36.26	0.164	35.47	^**^	11.0	2

^a^Mean root length; ^b^coefficient of variation calculated as ratio of the standard deviation to the mean; ^c^variance in root length; ^d^significance of variance relative to WT as determined by Levene’s test, ^*^, P < 0.05; ^**^, P < 0.01; ^***^; P < 0.001; ^e^reduction in root length relative to WT; ^f^fold up-regulation of *AtCHR23* relative to WT. WT, wild-type; na, not applicable; nd, not detected.

**Figure 2:**
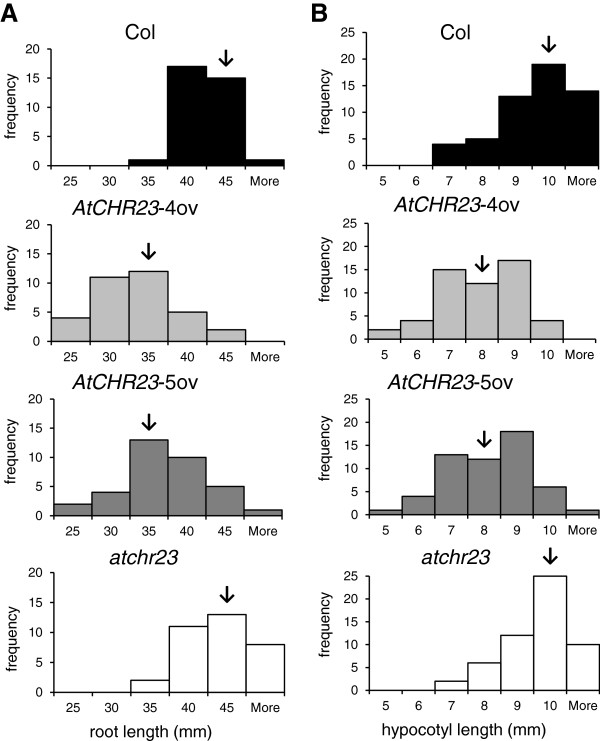
**Frequency distribution of root (A) and hypocotyl (B) length.** Seedlings (40 for each panel) were grown on agar plates for eight days at 23°C **(A)** or 28°C **(B)** in long-day conditions. In each panel, the arrow indicates the median length.

The variability in the phenotypic assays was assessed in more detail by analysis of the frequency distributions of the length data (Figure 2). The frequency distribution of the root lengths shows that the distribution is shifted to shorter roots when *AtCHR23* is over-expressed (Figure 2A), but still quite a number of individual seedlings have roots as long as the wild-type (Figure 2A, middle two panels). Also for the distribution of the hypocotyl length, the variation is larger in populations of over-expressing seedlings than in the wild-type (Figure 2B, middle two panels). In view of all experimental efforts to standardize the environment in the phenotypic assays, we think the variation between individuals of over-expressing lines is likely to have a molecular and/or functional basis.

To associate the growth arrest phenotypes with the level of *AtCHR23* mRNA, the amount of *AtCHR23* mRNA was determined in pools of (eight) seedlings with the help of qRT-PCR. The quantitative results are summarized in Table 1. A two-fold increase in *AtCHR23* mRNA (compared to wild-type) is observed in CHR23:*G*_*AtCHR23*ov. This is apparently sufficient for the growth arrest phenotype to become detectable. Higher levels of mRNA tend to make the phenotype more pronounced, without however a clear correlation between the level of up-regulation and the length of the root. Such an association indicates a complex interplay of interactions between steady-state mRNA levels and the penetrance of the root length phenotype. The lack of correlation between root length and the level of *AtCHR23* expression was also confirmed in individual seedlings of wild-type and mutant (10 seedlings of each) (data not shown).

### The reduction in root growth is due to reduced cell elongation

**Figure 3:**
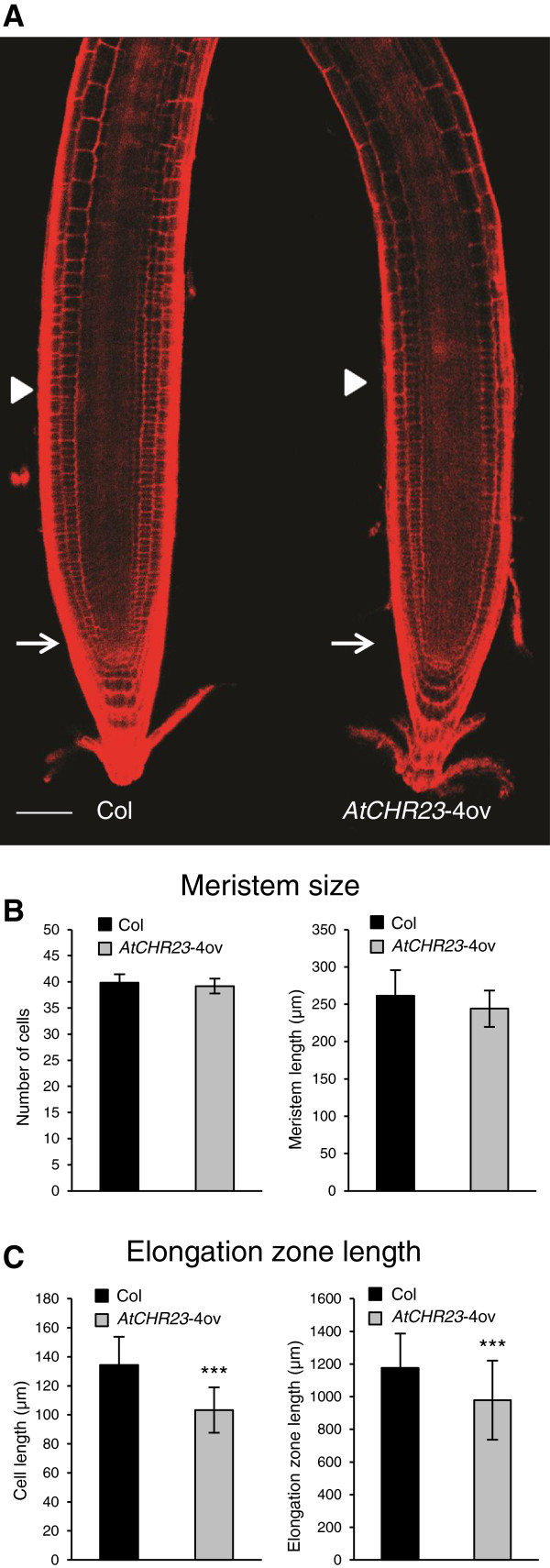
***AtCHR23 *over-expression affects cell elongation. (A)** Confocal images of 6-day-old Col wild-type and *AtCHR23*-4ov mutant roots grown at 23°C in long day conditions stained with propidium iodide. Arrows indicate the quiescent center, arrowheads indicate the boundary between the proximal meristem and elongation zone of the root. Scale bar: 50 μm. **(B)** Number of cells (± SD) counted in meristem (left) and mean (± SD) meristem length (right) in Col wild-type and *AtCHR23*-4ov mutant. **(C)** Mean (± SD) length of fully elongated cells in elongation zone (left) and mean (± SD) length of the elongation zone (right) in Col wild-type and *AtCHR23*-4ov mutant. Asterisks indicate significant differences from the wild type: ^***^, P < 0.001.

To determine whether the reduction of root length is due to reduced cell division or reduced cell elongation, we analyzed the size of the meristematic and elongation zone of 6-day-old seedlings. *AtCHR23*-4ov roots exhibited a normal cellular patterning compared to the wild-type (Figure 3A). For meristem we measured both the length of the meristematic zone and the number of meristematic cortex cells. None of them differ between wild-type and mutant roots (Figure 3B). To further assess the role of cell division, we also used the cell G2-M phase cycle marker pCYCB1;1:CYCB1;1-GUS [29]. No clear difference in the pattern (Additional file1: Figure S2) and number of GUS-positive cells was observed between the wild-type and the over-expressing mutant (data not shown). This is consistent with meristem size of wild-type and mutant (Figure 3B). On the other hand, the mutant showed a significantly shortened (16.8%) elongation zone relative to the wild-type as well as reduced length (23.1%) of the fully elongated cells (Figure 3C). Taken together, these results indicate that the major effect of *AtCHR23* up-regulation in the root is the reduction of cell elongation.

### Over-expression of *AtCHR23* results in smaller seedlings and smaller plantlets

**Figure 4:**
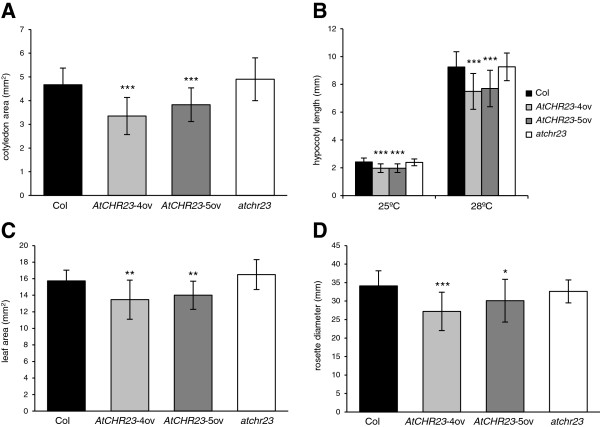
**Over-expression of *AtCHR23 *leads to overall reduced seedling and plant growth. (A)** Mean (± SD) cotyledon area of 8-day-old wild-type (Col) and mutant seedlings grown at 25°C in long day conditions. **(B)** Mean (± SD) of hypocotyl length of wild-type (Col) and mutant plants grown for 8 days at 25°C or 28°C in long-day conditions. **(C)** Mean (± SD) leaf area of first rosette leaf of 15-day-old soil grown wild-type (Col) and mutant plants in long- day conditions. **(D)** Mean (± SD) rosette diameter of 4-week-old wild-type (Col) and mutant plants grown as in **(C)**. For each line, 40 seedlings or 15 plants were measured. Asterisks indicate significant differences from the wild type: ^**^, P < 0.01; ^***^, P < 0.001.

Analyses of two *AtCHR23*-ov lines demonstrate that over-expression of *AtCHR23* also resulted in overall reduced seedling and plant growth (Figure 4). Over-expressing lines showed reduced growth of the cotyledon (Figure 4A) and hypocotyl (Figure 4B). The mean cotyledon area was reduced from 4.67 mm^2^ in the wild-type to 3.35 mm^2^ in *AtCHR23*-4ov (28.3% reduction) and to 3.83 mm^2^ in *AtCHR23*-5ov (18% reduction). The length of the hypocotyls was determined from seedlings grown at 25°C or 28°C. The latter temperature is known to induce considerable hypocotyl elongation [30]. The average hypocotyl length of 25°C-grown 8-day-old seedlings of over-expressing lines was reduced to 1.97 mm (about 20% reduction) compared to 2.42 mm of the wild-type, while the length of the hypocotyl of the knockout did not differ significantly from the wild-type. Such differences become more obvious at 28°C (Figure 4B). Both temperatures show that up-regulation of *AtCHR23* leads to a significant overall reduction in the growth of seedlings. The increased growth variability of mutants cotyledon and hypocotyl was not significant (Levene’s test; Additional file 2: Table S1).

To determine if and how the effects on plant size due to *AtCHR23* over-expression generate phenotypic changes further in development, two parameters for vegetative growth were measured in soil-grown plants: the leaf area and the diameter of the rosette. Both parameters were determined from digital images of 15 soil-grown plants. The average surface area of the first rosette leaf of the wild-type was 15.7 mm^2^. This was reduced to 13.5 mm^2^ in *AtCHR23*-4ov and to 14.0 mm^2^ in *AtCHR23*-5ov, so over-expressing lines have up to 15% smaller leaves than the wild-type (Figure 4C). The knockout line had slightly larger leaves (5%), but again this difference was not statistically significant in the experimental set-up chosen. Similar growth differences were observed for the third rosette leaf (data not shown). Leaves of over-expressing mutants also showed significantly increased growth variability relative to wild-type (Levene’s test; Additional file 2: Table S1). Furthermore, the average rosette diameter of 4-week-old over-expressing mutants was reduced in size (Figure 4D). While the wild-type rosette diameter was 34.1 mm, it was 27.2 mm in *AtCHR23*-4ov and 30.1 mm in *AtCHR23*-5ov. Compared to the wild-type it represents 20% and 12% reduction in the size of the rosette in the mutants, respectively. It shows that also during vegetative development plants over-expressing *AtCHR23* tend to stay smaller than the wild-type.

### Light conditions determine the growth characteristics of over-expressing lines

**Figure 5:**
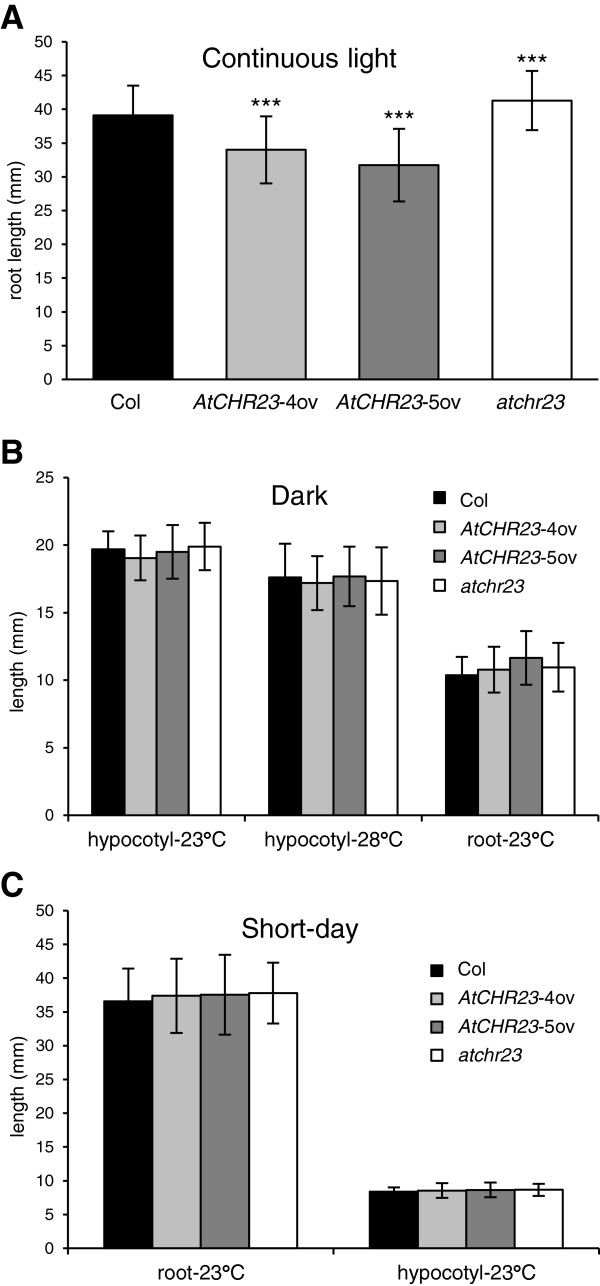
***AtCHR23 *over-expression only affects root length in sufficient light. (A)** Mean (± SD) root length of wild-type (Col) and mutant seedlings grown for 10 days at 23°C in continuous light. **(B)** Mean (± SD) root and hypocotyl length of 10-day-old wild-type (Col) and mutant seedlings grown at the indicated temperature in the dark. **(C)** Mean (± SD) root and hypocotyl length of 10-day-old wild-type (Col) and mutant seedlings grown at 23°C in short-day conditions. For each line 40 seedlings were measured. Asterisks indicate significant differences from the wild type: ^***^, P < 0.001.

As light is a crucial environmental factor affecting plant growth [31], we evaluated the growth dynamics of the various *AtCHR23* expression variants under different light regimes. In continuous light, all *AtCHR23* mutants confirm the pattern of root length as presented above for long-day conditions. Over-expressing lines have a significantly reduced root length relative to the wild-type, whereas the knockout tends to have (in this case indeed significantly) longer roots (Figure 5A). In the dark, however, none of the lines significantly differed in root length from that of wild-type (Figure 5B). In the dark, root growth is known to be significantly reduced [32,33], while the hypocotyl is known to elongate (etiolate) more than in the light [34]. Establishing further reductions in root length in such an environment is therefore less reliable. However, also the length of the hypocotyl of seedlings grown in the dark at either 23°C or 28°C (Figure 5B) was not different from the wild-type. Also at short day conditions (10 days at 8 h light/16 h dark at 23°C; Figure 5C), the length of neither roots nor hypocotyls of mutants could be distinguished from the wild-type. One possible cause for the lack of the phenotype in dark and short-day could be the instabilities of *AtCHR23* mRNA over-expression. However, quantitative expression analysis of *AtCHR23* in dark and short-day grown seedlings confirmed the same level of up-regulation relative to wild-type as in long-day (data not shown). The lack of phenotype in dark and short-day grown mutants cannot be therefore explained by reduced levels of *AtCHR23* over-expression. These results show that light markedly influences the impact of modified *AtCHR23* expression on the growth dynamics of Arabidopsis seedlings: sufficient (amounts of) light is required to establish the *AtCHR23*-mediated growth phenotype.

### Abiotic stress magnifies the impact of *AtCHR23* over-expression

**Figure 6:**
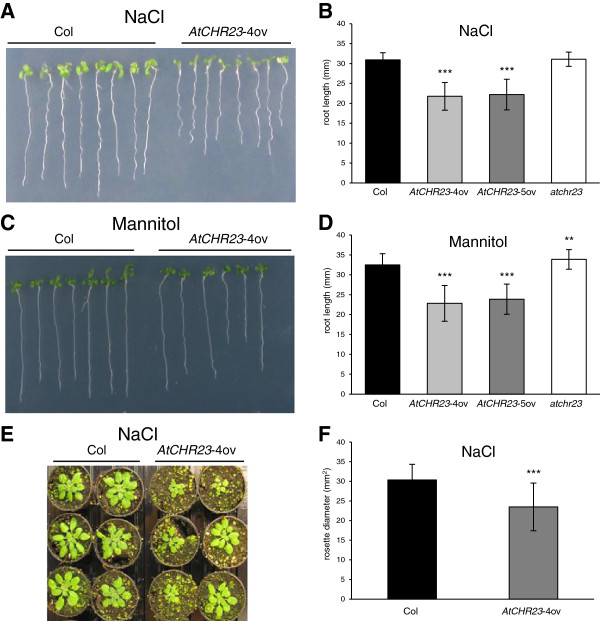
**Abiotic stress emphasizes the reduction of growth in case of *AtCHR23 *over-expression. (A)** Photograph of 8-day-old seedlings grown at 23°C in long-day conditions on medium supplemented with 75 mM NaCl. **(B)** Mean (± SD) length of the primary roots of 8-day-old seedlings grown on 75 mM NaCl. **(C)** Photograph of 8-day-old seedlings grown at 23°C in long-day conditions on medium supplemented with 200 mM mannitol. **(D)** Mean (± SD) length of the primary roots of 8-day-old seedlings grown on 200 mM mannitol. **(E)** Photograph of 4-week-old wild-type and *AtCHR23*-4ov plants two weeks after application of salt stress. **(F)** Mean (± SD) rosette diameter of 4-week-old plants two weeks after application of salt stress. For each assay and line, 40 seedlings or 15 plants were measured. Asterisks indicate significant differences from the wild type: ^**^, P < 0.01; ^***^, P < 0.001.

The impact of modified *AtCHR23* expression is also apparent in environmental stress. Seedlings were assayed under abiotic stress conditions on agar plates containing 75 mM NaCl (salt stress; Figure 6A) or 200 mM mannitol (osmotic stress; Figure 6C). Both stresses had, as expected, a clear negative impact on root growth. The average length of the roots of wild-type seedlings in an environment with salt stress was 30.92 mm (Figure 6B) and in osmotic stress 32.51 mm (Figure 6D), whereas without such stress the length was 40.7 mm (see Table 1 and Figure 1). This shows that salt stress reduces the root length of the wild-type by 24% and osmotic stress by 20%. The over-expressing mutants *AtCHR23*-4ov and *AtCHR23*-5ov respond to salt by 32% and 36% reduction of root length, respectively (Figure 6B). In osmotic stress, this reduction was 29% and 31%, respectively (Figure 6D). Similar results were obtained with the lines over-expressing *AtCHR23* cDNA copy (Additional file 1: Figure S3). In contrast, the knockout line *atchr23* has slightly longer roots than the wild-type*,* but only in osmotic stress (average length 33.9 mm; Figure 6D). These data indicate that the *AtCHR23* over-expressing lines respond to stress conditions by stronger growth arrest of the root length than the wild-type. A non-parametric factor analysis showed highly significant (P < 0.001) effects of both genotype and stress treatment on root length, and significant (P < 0.01) effects of genotype X treatment interaction on root length, in all mutant lines except for knockout line at osmotic stress (Additional file 2: Table S2). The same is observed in further vegetative development. After applying salt stress by watering two-week-old plants with 100 mM NaCl twice in 3 days, the rosette diameter of soil-grown plants (Figure 6E) was measured. The rosette diameter of wild-type without stress was 34.1 mm^2^ whereas after stress, it was 30.34 mm^2^, which is a reduction of 11%. The *AtCHR23*-4ov plants respond to salt stress by two-fold higher (22%) reduction of the rosette diameter: from 30.1 mm^2^ to 23.49 mm^2^ (Figure 4D, 6F). The non-parametric factor analysis showed highly significant (P < 0.001) effects of both genotype and treatment on rosette diameter, however the effect of genotype X treatment interaction was not significant (Additional file 2: Table S2). It shows that abiotic stress magnifies the effect of *AtCHR23* over-expression on the seedlings growth and that the effect extends beyond the seedling stage.

### Genome-wide RNAseq analysis demonstrates increased variability of gene expression upon *AtCHR23* over-expression

The growth phenotype conferred by *AtCHR23* over-expression was evaluated by RNA sequencing. Two biological replicates of pooled eight-day-old seedlings of *AtCHR23-*4ov and the wild-type (Columbia) grown at 23°C in long-day (with the reduced growth phenotype) and short-day (without the reduced growth phenotype) photoperiods were evaluated. For each of the eight samples, more than 60 million reads were generated. Given the experimental set-up, expression differences associated with the reduced growth phenotype were expected between the over-expressing line in long-day conditions relative to all other samples.

**Figure 7:**
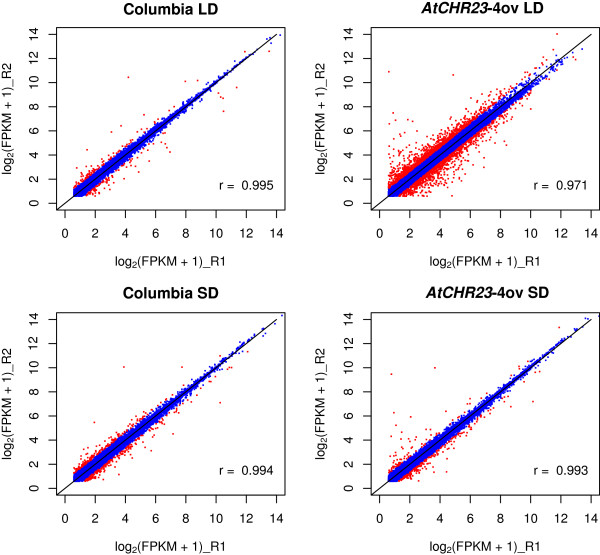
**Scatter plots of gene expression expressed as log**_**2**_**(FPKM + 1) show more pronounced variability in long-day grown over-expressing mutant.** Expression was determined from RNAseq reads for the wild-type (Columbia) and mutant (*AtCHR23-*4ov), with biological replicates indicated with R. Each dot represents a gene. Genes displaying a variability of expression above the cut-off specified (see text) are shown in red. In the bottom of each graph the pair-wise Pearson’s correlation of all genes depicted is shown. LD, long-day; SD short-day; R1, biological replicate 1; R2, biological replicate 2.

Differential expression analysis using DESeq [35] or cuffdiff [36] resulted in lists of potentially differentially expressed (DE) genes. However, in additional biological replicates many of these could not be confirmed. From 96 genes identified by DESeq as potentionally DE in long-day mutant (Additional file 3), 24 genes were analyzed by qRT-PCR and 7 were confirmed as differentially expressed (33.3% of tested genes). We concluded that identified DE genes cannot be biologically validated. Further analyses therefore focused on the apparent variation in gene expression. Comparison of the expression values expressed as summed fragments per kilobase of transcript (exon model) per million mapped reads (FPKM) of replicates R1 and R2 for each sample showed the Pearson’s correlation coefficients above 0.99 (Figure 7), except for the only sample in which the growth phenotype was present: *AtCHR23* over-expression in long-day conditions. In this case the data are much more disperse from the line of best fit and the Pearson’s correlation coefficient is just above 0.97 (Figure 7). In order to assess the larger between-replicate expression variability in mutant long-day, we calculated for all genes the absolute differences between the log_2_(FPKM + 1) expression level in the two replicates. The larger expression difference shown by the top 1% of the genes in wild-type (195 genes) was taken as cut-off for variability and used to select the number (and identity) of the genes in all other samples that showed variability higher than specified cut-off. This threshold was equivalent to an expression difference of about 1.5 fold on the normal scale. In the scatter plots of genome-wide gene expression, these genes are depicted in red (Figure 7).

**Figure 8:**
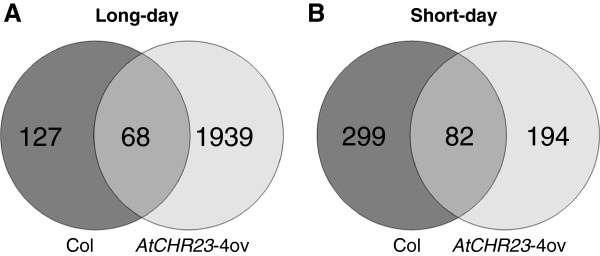
**Numbers of genes with high expression variability.** The number of plant-line specific and shared genes that are identified as variable are given for the wild-type (Col) and over-expressing mutant (*AtCHR23*-4ov) in long-day **(A)** and short-day **(B)** conditions.

**Figure 9:**
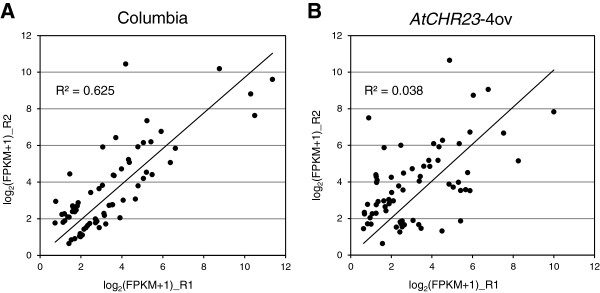
**Over-expression of *AtCHR23 *pronounces the variation in genes that are already variable in the wild-type.** The panels show scatter plots and the linear regression line of the gene expression expressed as log_2_(FPKM + 1) of the 68 genes identified as variable of the two biological replicates of **(A)** the wild-type (Columbia) and **(B)** the *AtCHR23*-4ov over-expressing line, both grown in long-day conditions. The coefficient of determination (R^2^) is shown in the panel. R1, biological replicate 1; R2, biological replicate 2.

In long-day conditions, the *AtCHR23* over-expressing mutant has no less than 2007 genes with larger variation (Figure 8A). Of these, 68 genes were also variable in wild-type (Figure 8; Additional file 4). This shows that *AtCHR23* over-expression increases the expression variability of a considerable subgroup of genes compared to the wild-type. In contrast, in short-day conditions, 381 genes were identified as variable in the wild-type, whereas 276 genes were identified in the mutant line, of which 82 were shared (Figure 8B; Additional file 4). The larger subgroup of variable genes is therefore associated with the higher over-expression of *AtCHR23* observed in long-day conditions. This may point to a causal relationship between *AtCHR23* over-expression and increased variability of gene expression. The 68 long-day variable genes shared between the wild-type and the mutant are less correlated between the two replicates of *AtCHR23* over-expressing mutant (R^2^ = 0.038) relative to the wild-type (R^2^ = 0.625) (Figure 9). It indicates that the expression of genes which are already noisy in natural conditions (the wild-type) become even more noisy when *AtCHR23* is over-expressed.

To evaluate the function of the genes with higher variation in gene expression when *AtCHR23* is over-expressed*,* gene ontology (GO) analysis was performed. For this, the subset of 298 genes (from the 2007) was selected that had at least 3-fold expression difference between the two biological replicates. Genes were classified using the Classification SuperViewer [37] as being over- or under-represented. The main results are summarized in Additional file 1: Figure S4. Biological Process subcategories that were over-represented include responses to stress, stress stimuli and developmental processes, in addition to other biological processes. This is in good agreement with the phenotypic observations presented above.

### *AtCHR23* over-expression enlarges differences in gene expression among individuals for selected subsets of genes

**Figure 10:**
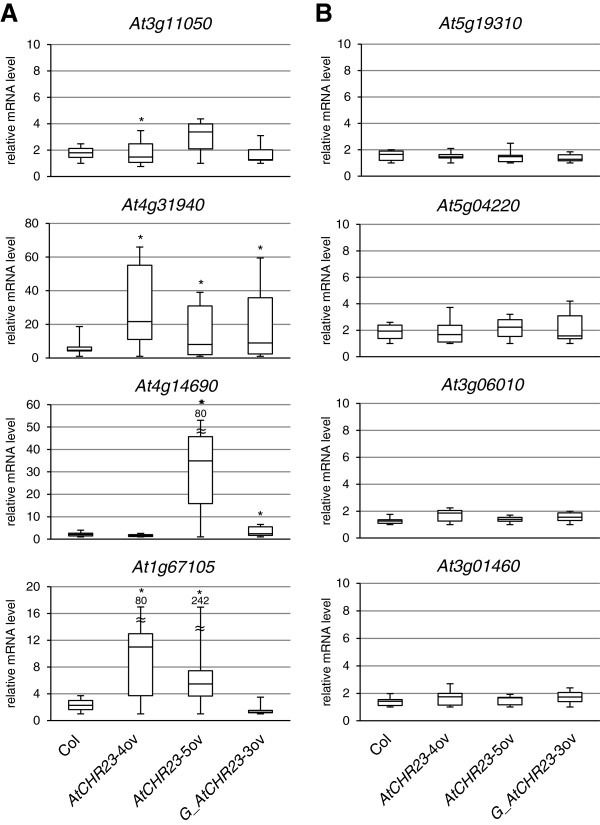
**Box plots of the relative mRNA levels of selected genes in individual seedlings.** Relative mRNA levels were determined in six individual seedlings, each of wild-type (Col) and three *AtCHR23* over-expressing lines, by quantitative RT-PCR using gene specific primers for eight different genes. Four of these genes **(A)** were characterized by high variability between the replicates of the over-expressing lines and four genes **(B)** did not show such variability. After normalization to the *UBC* reference gene, the expression value for each gene and each individual seedling was scaled relative to the lowest expression value (set to 1) for that gene in the line. In each box plot, the box area represents the lower and the upper percentiles, the horizontal line within the box indicates the median value and the horizontal dashes at the top and the bottom of the box show the minimum and maximum values observed. Asterisk indicate significantly different variances (Levene’s test) from the wild type at P < 0.05.

To address the impact of variation on gene expression in individual seedlings, eight genes were selected for additional analyses. Four genes were randomly selected from the list of *AtCHR23-*4ov variable genes at long-day conditions. In addition four genes were randomly selected that were identified as not variable (including *AtCHR23*/*At5g19310*). Details of these genes are given in Additional file 2: Table S3. The expression of these eight genes was analyzed by quantitative RT-PCR in six individual seedlings of over-expressing mutants and the wild-type grown at long-day conditions. Box plots summarizing these data show considerably more variation in expression among individual seedlings of the various mutants compared to the wild-type for the four variable genes (Figure 10A). In contrast, none of the genes selected for lack of variation showed such a large expression variability between individual seedlings in any of line tested (Figure 10B). In individual seedlings different from seedlings analyzed in Figure 10 additional three variable (*At1g04220*, *At3g22640*, *At3g12580*) and three not variable genes (*At5g02490*, *At5g10140*, *At2g01422*) were analyzed. For all of them, except for *At3g12580*, the variability as detected by RNAseq was confirmed. Although based on limited number of individuals these data show that the increased variation of gene expression of distinct subset of genes is also apparent in individual seedlings. This emphasizes the importance of studying the expression pattern in individual plants. For direct biological proof of increased expression variation in *AtCHR23* over-expressing mutant more genes should be tested, preferably by extensive RNAseq analysis of a larger number of individual seedlings.

## Discussion

Previously, we have presented detailed analyses of the over-expression of *AtCHR12*[7]. *AtCHR12* is a paralog of *AtCHR23*: the two genes are very similar in (protein) sequence and are thought to be result of recent gene duplication in Arabidopsis [27]. Yet, their expression patterns are different in spatial and quantitative terms [38] and the results presented here show that over-expression of these two genes have different impact on the growth parameters of Arabidopsis. Over-expression of *AtCHR12* had not the phenotypic effects here documented for *AtCHR23* over-expression. Under normal environmental conditions, the phenotype of the *AtCHR12* mutant cannot be distinguished from the wild-type. However, exposing the *AtCHR12* over-expressing mutant to mild stress conditions led to growth arrest of normally active primary buds and reduced growth of the primary stem. It demonstrated that chromatin remodeling-associated growth arrest is priming the plants for growth arrest upon actual stress after the transition to reproductive development [7].

*AtCHR23* over-expression affects the growth of seedlings and the vegetative rosette. As the two ATPases affect plant growth in different ways at different stages of development, these two genes present a clear and not-so-common example in Arabidopsis of sub-functionalization of very similar genes after gene duplication [38]. In the genome of crops as potato and tomato, only a single gene instead of two paralogs is present [27]. This single ortholog is supposed to combine the function of both *AtCHR12* and *AtCHR23* in the environmental growth response of such crops. If confirmed in future experiments it could open a new possibility to improve the environmental growth response of agriculturally relevant crops.

### Over-expression rather than loss-of-function is required to observe the growth phenotype

The *atchr23* loss-of-function mutant did not show any visible phenotype, neither did the equivalent *atchr12* loss-of-function mutant. The *atchr12*/*atchr23* double knockouts fail to initiate root and shoot meristems and are embryo lethal (own observations; [19]). A barely viable double mutant containing weak knockout alleles showed severe defects in the maintenance of stem cells, extremely delayed development, bushy appearance, floral organ aberrations and substantially shortened roots [19]. The short roots of such double knockouts had significantly reduced cell division and other defects in meristem organization [19]. It suggests that in embryo and endosperm development, these two paralogous genes seem to be redundant [19]. In contrast to the phenotypic impact of the weak double knockout, *AtCHR23* over-expression is not associated with any morphological effect on the root meristem (Figure 3A). Over-expression correlates with a shorter root elongation zone and a reduced hypocotyl and cotyledon growth. The post-embryonic growth of these organs is mostly the result of controlled cell elongation and does not involve significant cell divisions [34,39]. Therefore, *AtCHR23* over-expression exerts (most) impact on cell expansion rather than on cell division. The phenotype of the weak double knockout was accompanied by the changes in the expression of several meristem marker genes (*WOX5, SHR*, *SCR*) and several cell cycle genes [19]. None of these genes was identified as differentially expressed and/or variable in the RNAseq analysis of the *AtCHR23* over-expression phenotype here presented.

Over-expression accomplished by either the native or the much stronger CaMV 35S promoter resulted in the same growth phenotype. A relatively small fold up-regulation of about 2 (Table 1; line CHR23:*G*_*AtCHR23*ov) is apparently sufficient and no clear correlation between fold up-regulation and root length reduction is evident (Table 1). This lack of dose dependency of up-regulation indicates that other limiting factors are likely to be involved. SWI/SNF2 ATPases function in the context of protein complexes [40] and one of the participants of such a complex may become limiting. The larger amount of ATCHR23 may be in competition with such a factor, or drains it from such complexes. If so, the over-expression phenotype would represent an on/off case and allow for more fine-grained analysis of the detailed role of ATCHR23 in growth regulation than the analyses of knockouts [41]. Such severe effects from small folds of up-regulation are rather difficult to study, but especially in a complex phenotype as vegetative development, such subtle effects may be more the rule than the exception [42]. Several other examples are known where relatively small fold over-expression results in clear phenotypic effects [43,44].

### ATCHR23 function needs long-day light condition

The growth phenotype of *AtCHR23* over-expression is only apparent in (sufficient) light. Its function or the function of (one of its) direct partners must therefore be photoperiod-dependent. Photoperiodicity is one of the most significant and complex of interactions between plants and their environment. It is the major stimulus that plants use to detect seasons [45]. A well-known response to the photoperiod is flowering, but it also affects seed germination, leaf formation rate, leaf size and dry matter production [46]. The lack of a clear phenotype in dark and short-day condition could result from dark-induced proteolytic degradation of either ATCHR23 or ATCHR23 targets. An example of such a regulation is the transcriptional regulator CONSTANS (CO) that promotes flowering of *Arabidopsis thaliana* under long summer days, but not under short winter days [47]. More detailed analyses of ATCHR23 will be required to assess whether protein stability plays a role in the regulation of and/or by *AtCHR23.* Conversely, the putative target genes of ATCHR23 may be light regulated and the ATCHR23 remodeling complex contributes to the fine-tuning of this regulation. In view of the reaction of the various plant lines to environmental stress (Figure 6), light or light duration may become perceived as stress.

### Over-expression of *AtCHR23* increases variability of growth and gene expression

The length of the root (or the hypocotyl) in populations of seedlings behaves as a typical quantitative trait: it has a frequency distribution around a population average of in this case genetically identical plants. Over-expression of *AtCHR23* shifts the distribution to an average of shorter roots (Figure 2A) and hypocotyls (Figure 2B). Such a continuous distribution indicates a polygenic trait rather than a phenotype controlled by a single locus and/or the involvement of the environment in which epigenetic processes are believed to play an important role [48,49]. The frequency distributions tend to overlap and when considered on an individual basis, some over-expressing seedlings are individuals with growth comparable with individual wild-type seedlings (Figure 1A, Figure 2). In the individual case, over-expression of *AtCHR23* may not necessary result in reduced growth. This indicates that over-expression does not have inhibitory effect on growth *per se*. Upon over-expression, the mutant seedlings show a more broad distribution of growth parameters than the wild-type (Figure 2). Therefore, *AtCHR23* over-expression increases the within-population variability of growth, best expressed as coefficient of variation (CV; Table 1) of growth*.*

In long-day conditions, *AtCHR23* over-expression associates with increased variability of gene expression between biological replicates. It is tempting to assume a causal relationship between these two associated phenomena. Although the larger variation could originate from environmental factors and/or the biological material used (seed batches), extreme care was taken to exclude interference of such experimental factors. Light intensity, temperature, humidity were carefully controlled and monitored; wild-type and mutant seedlings were without exception grown at the same time and the same conditions; RNA samples were isolated simultaneously and RNA handling procedures were synchronized as much as possible. Moreover, the variability of the same over-expressing line in short-day conditions was similar to that of the wild-type.

The apparent effect of *AtCHR23* over-expression on increasing expression variability is not unknown in other biological systems. Inter-individual differences in gene expression is observed in many organisms, including human, mice, fish and yeast [50-52]. Also in plants, considerable variability of gene expression can occur between genetically identical individuals in identical environments. Gene expression can differ seemingly randomly in amplitude, frequency and timing between genetically identical cells. Such stochasticity of gene expression is nongenetic or epigenetic in nature and thought to be an intrinsic property of gene expression itself: stochastic noise [53]. The 68 genes identified as variable in both the wild-type and the over-expressing mutant could represent intrinsically variable or noisy genes in the tissues and conditions examined. Because *AtCHR23* over-expression makes intrinsically variable genes more variable, the ATCHR23 remodeling complex could be involved in tuning the noise levels of variable genes. Stochastic noise can be beneficial, *e.g.* for survival in fluctuating stressful environment [54,55], but in general it is considered to decrease fitness or interfere with development [53,56].

Chromatin-related events are thought to be a component of the regulation of the stochastic noise in gene expression. For example, in yeast, deletion of individual components of chromatin remodeling complexes such as SWI/SNF increased the expression fluctuation from the PHO5 promoter significantly [55] and variable genes are distinctly regulated by chromatin modifiers [57]. In most biological systems known today, the intrinsic variability of expression has to be controlled or buffered [58] to ensure optimal development and growth. The ability to buffer variations generated by molecular noise, or environmental fluctuations is termed robustness [20]. It is suggested that the expression of genes with an essential role in development or differentiation is highly robust [59], whereas expression of stress-responsive genes tends to be much more variable between cells and individuals [53,60]. The latter suggestion is in agreement with the significant enrichment for stress and stress stimuli responsive genes in the GO analyses of genes that are highly variable between the two replicates of the *AtCHR23* over-expressing line grown in long-day conditions (Additional file 1: Figure S4).

### The putative perils of pooling

The best way of RNAseq analysis is still being discussed [61,62] and may depend on both biological and statistical issues, such as sampling, pooling, pooling design, the distribution of (biological or environmental) variation and others [63,64]. Accounting for biological variation in gene expression is important for reliable and biologically relevant differential expression analysis [65]. Large variation of the expression of subset of genes between the individual seedlings in pools (Figure 10) could for example result in poor reproducibility between data from different pools.

We have here presented an RNAseq data analysis that is focussing on the variability of gene expression as the topic-of-interest. Independent validation by qRT-PCR showed the validity of the approach developed, although more advanced statistics may distill more understanding from the RNAseq data here presented. This data is based on pools of genetically identical seedlings that however may show highly variable gene expression. Such between-individual variation in gene expression did not yet get too much attention, but the depth of the new sequencing technologies could provide approaches to circumvent this limitation [66]. In fact, also the analysis of a single whole seedling concerns a pool of various tissues in different developmental stages that may have differences in gene expression. In the future, large-scale single cell transcriptomics may resolve such complexities [67,68].

## Conclusions

We have shown that in transgenic Arabidopsis, the over-expression of the SWI/SNF2-type ATPase *AtCHR23* increases the variability of growth and the variability of expression of a distinct subset of genes in populations of genetically identical plants. These results suggest that accurate and controlled expression of *AtCHR23* contributes to more stable or robust gene expression that results in a more uniform growth phenotype. Based on the phenotypic and expression data here presented we propose that the ATCHR23 remodeling complexes could be a component of a buffering system of gene expression in plants. If that system of buffering is disrupted by over-expression of *AtCHR23,* downstream genes become more variable and compromise the expression of other genes in ways that result in the reduced growth phenotype here documented. Phenotypic robustness influences all parameters important for plant growth, yield and quality. The findings presented here will help to better understand and use chromatin remodeling genes as exponents of a potential buffer of phenotypic and transcriptional variation, particularly in conditions of changing environments.

## Methods

### Construction of T-DNA plasmids for transformation

To generate plants that over-express *AtCHR23* the genomic copy of the gene sequence (including all 11 introns) was obtained by PCR from Arabidopsis Col-0 wild-type. Three sets of primers were used with the Phusion™ DNA polymerase (see Additional file 2: Table S4 for details). All three PCR fragments were cloned into pJET (Fermentas) and verified by sequencing. The genomic copy of *AtCHR23* was assembled by ligation of appropriate restriction fragments of three PCR sequences into pENTR4 (Invitrogen). The resulting plasmid carries the whole gene including 127 nucleotides of 5’UTR. This was recombined in an LR Gateway (Invitrogen) reaction with pB2GW7 (http://gateway.psb.ugent.be/). The resulting binary vector 35S::*AtCHR23* (Additional file 1: Figure S1) was introduced into *Agrobacterium tumefaciens* C58C1 (pMP9) and used for Arabidopsis transformation. Transgenic lines were selected based on PPT resistance (5 μg ml^-1^ phosphinothricin-DL) and screened for the level of transgene expression. Such lines were designated *AtCHR23*-ov. In addition, two GFP-tagged constructs carrying a cDNA copy of *AtCHR23* driven either by the CaMV 35S or the endogenous promoter were prepared (Additional file 1: Figure S1). The cDNA copy of *AtCHR23* was prepared from RNA with the SuperScript^®^ III First-Strand Synthesis System employing the oligo(dT)_20_ primer (Invitrogen) and PCR amplification using the CHR23_F4 and CHR23_R4 primers (Additional file 2: Table S3). The full length cDNA sequence was recombined by Gateway BP clonase into the pDONR221 entry vector (Invitrogen). The resulting plasmid was next recombined in an LR Gateway reaction into the destination vector pK7FWG2 (http://gateway.psb.ugent.be/). The resulting binary vector 35S::*GFP*-*AtCHR23* was used for Arabidopsis transformation. Transgenic lines were selected based on kanamycin resistance (50 μg ml^-1^) and the level of transgene expression. Such lines were designated *G*_*AtCHR23*-ov. The promoter sequence (918 bases) of *AtCHR23* including the 5 ‘UTR was isolated by PCR using the primers pCHR23_F and pCHR23_R (Additional file 2: Table S4) and cloned into the pENTR4 entry vector. The desired promoter sequence was selected with appropriate restriction enzymes and cloned into a derivative of pENTR4 carrying the *GFP* gene. The resulting clone was, together with *AtCHR23* cDNA entry clone described above, assembled in a multi-step LR Gateway reaction into the modified destination vector pBGW (http://gateway.psb.ugent.be/). The resulting binary vector pCHR23::*GFP*-*AtCHR23* was transformed to Arabidopsis. Transgenic plants were selected based on PPT resistance (5 μg ml^-1^ phosphinothricin-DL) and the line used for further analysis was designated CHR23:*G*_*AtCHR23*ov.

### Plant material and growth conditions

All transgenic Arabidopsis plants over-expressing *AtCHR23* were generated by transformation of wild-type *Arabidopsis thaliana* Col-0 using the floral dip method [69]*.* For analysis, homozygous F3 plants were used. The loss-of-function mutant lines of *AtCHR23* (*At5g19310*) SALK_057856 and SALK_139883 were obtained from the Arabidopsis Stock Center (Salk Laboratory, Institute of Genomics Analysis, USA; generated by J.R. Ecker [70]). SALK_057856 carries the T-DNA insertion in the first exon and SALK_139883 carries T-DNA in the fifth exon of *AtCHR23*. The zygosity of both SALK lines was determined on 30 μg ml^-1^ kanamycin plates. For both knockouts no full length cDNA product was detected (data not shown). The marker line pCYCB1;1:CYCB1;1-GUS [29] in Col-0 was obtained from M. Koornneef (Cologne/Wageningen). In all cases seeds were stratified for 3 days at 4°C in the dark before sowing or analysis to synchronize germination. Seedlings were grown vertically in fully controlled growing chambers lit by Philips TD 32 W/84HF lamps at either 23°C, 25°C or 28°C in long-day (LD; 16 h light/8 h dark) or short-day (SD; 8 h light/16 h dark) photoperiods. Light conditions were adjusted according to the experimental set-up. Plants were grown in standard potting soil in 16 h light/8 h dark (long-day conditions) at 21 ± 2°C in either a growth room lit by Philips-Master 36 W/830 lamps or in a controlled greenhouse with supplemental light provided by four Son-T (Philips Greenpower, 400 W) lamps when required.

### Analysis of growth parameters

Root elongation assays were performed as described [7] on seedlings grown vertically on 0.5 × MS agar plates. For salt or osmotic stress treatments, seedlings were grown on plates supplemented with 75 mM NaCl or 200 mM mannitol, respectively. Seedlings were photographed (Canon SX120) after 8–10 days of growth and the root length was measured from the root tip to the base of the hypocotyl using ImageJ (http://rsb.info.nih.gov/ij). Detached hypocotyls, cotyledons and leaves were flattened on double-sided tape and also photographed for analysis with ImageJ. For the analysis of vegetative rosette growth, plants were photographed 4 weeks after germination, just before the transition to flowering. The diameter of the rosette was estimated using ImageJ after enclosing the entire rosette in a rectangular selection. The size of the meristem and elongation zone was determined in 6-day-old seedlings grown vertically on 0.5 × MS agar plates. The meristematic zone was measured as the length from the quiescent center till the transition zone and as the number of cells in cortex file between the quiescent center and the first cell of the transition zone. In the elongation zone was analyzed the length as the distance from the transition zone till the beginning of the differentiation zone and the size of the fully elongated cell. Images were obtained with Leica microscopes (Leica Microsystems) and were used in ImageJ for length determination. For most of the growth parameters, at least two to three replicates were performed. For measurements 15–20 roots were used. GUS patterns were observed as described previously [7] with Nikon Optiphot-2 microscope.

### Statistical analysis

For statistical analysis, normality of data was evaluated with Shapiro-Wilk test [71] and homogeneity of variances was tested with the Levene’s test [72] (http://www.stat.ufl.edu/~winner/sta6166.html). The significance of the difference between the means of wild-type and mutants in the same growth condition were calculated by non-parametric Mann–Whitney U test. For comparison of different growth conditions a non-parametric adjusted rank transform test [73] was used. In charts and tables, asterisks ^*^, ^**^, and ^***^, respectively, indicate significance at the 0.05, 0.01 and 0.001 of confidence levels.

### Analysis of gene expression by qRT-PCR and RNAseq

Seedlings were grown on agar plates in the same set-up and conditions as used for growth measurements (see above). Total RNA was isolated from the pools of eight intact 8-day-old seedlings using the E.Z.N.A.™ Plant RNA Mini Kit (Omega Bio-Tek, Inc., USA), followed by on column DNase treatment (Qiagen, RNase-free DNase Set). One microgram of RNA was used for cDNA synthesis using the iScript™ cDNA Synthesis Kit (Bio-Rad Laboratories, Inc., USA). Ten times diluted cDNA was used for quantitative RT-PCR using the iQ™ SYBR^®^ Green Supermix (Bio-Rad Laboratories, Inc., USA) in an iCycler thermal cycler. Reactions were performed in triplicate. The *UBC* gene (*At5g25760*) was used as reference [74]. Sequences of primers used are given in Additional file 2: Table S3.

For RNA sequencing, total RNA was isolated as above from eight 8-day-old seedlings of either mutant (*AtCHR23*-4ov) or the wild-type, grown at 23°C in either long-day (with the reduced growth phenotype) or short-day (without the reduced growth phenotype) photoperiods. In all cases, two biological replicates were included from two different seed stocks, either one year old (biological replicate 1) or half a year old (biological replicate 2). With four different conditions each with two biological replicates a total of 8 samples were analyzed. All eight RNAseq library preparations were performed according to manufacturer’s recommendations (Illumina Truseq RNA sample Preparation Low Throughput protocol). The eight samples were multiplexed in one Hiseq 2000 lane (WUR sequencing facility) and sequenced in 100 bases paired-end reads with an insert size of approximately 300 bases. After demultiplexing, for each of the eight samples, more than 60 million reads were generated.

### RNASeq bioinformatics

The quality of reads was assessed with FastQC (obtained from http://www.bioinformatics.babraham.ac.uk/projects/fastqc/). Adapter and quality trimming was performed with the CLCbio Genomics workbench (v. 5.5.1) using default settings. Reads were mapped against the *Arabidopsis thaliana* genome (v. TAIR10) using TopHat (v. 2.0.5; [75]) with as default parameter settings: --no-mixed, --no-discordant, -M, -g 1, --min-intron-length 50, --max-intron-length 11000. Differential expression was analyzed with DEseq (v1.10.1; [35]) and with cuffdiff in the cufflinks package (v. 2.0.2; [36]) using setting options –u and –b without quality trimming. Gene expression levels were determined by calculating the FPKM (Fragment per Kilobase of transcript (exon model) per Million mapped reads) values. To analyse the variation in expression, the expression levels between two replicates for each sample and conditions were compared for all genes with FPKM > 0.5 in both replicates. The absolute difference of the log_2_ transformed FPKM values [log_2_ (FPKM + 1); approximately equivalent to fold change on the normal scale] was calculated and the top 1% of the genes of the wild-type plants grown in long-day conditions was used to define a cut-off for all other conditions to determine the number (and identity) of genes with a difference (*i.e.* variation) larger than this cut-off. Gene ontology (GO) analysis was performed with the Classification SuperViewer tool [37] from the Bio-Array Resource (http://bbc.botany.utoronto.ca/ntools/cgi-bin/ntools_classification_superviewer.cgi). Genes were functionally classified according to the three main GO categories: biological process, molecular function, and cellular component.

## Competing interests

The authors declare that there are no competing interests.

## Authors’ contributions

AF (conducted the experiments, analyzed data, wrote manuscript); EIS (performed gene expression analyses, wrote part of manuscript); JK and HG (assisted with expression analysis); JV (generated constructs); JPN (assisted with interpretation of data and wrote manuscript); LM (designed experiments, analyzed data, wrote manuscript). All authors read and approved the final manuscript.

## Supplementary Material

Additional file 1: Figure S1 Schematic layout of T-DNA regions of plasmids used to generate transgenic Arabidopsis over-expressing *AtCHR23* gene. 35S, CaMV 35S promoter; pCHR23, *AtCHR23* promoter; *GFP*, green fluorescent protein gene; Kan, kanamycin resistance gene; BAR, barnase herbicide resistance gene; RB, LB, right and left T-DNA borders. Grey shaded box indicate the presence of 5’UTR. **Figure S2.** Photograph of whole-mount, GUS-stained 4-day-old roots of CYCB1;1:CYCB1;1-GUS in wild-type (left) and in *AtCHR23*-4ov homozygous for both transgenes. *AtCHR23*-4ov was crossed with the transgenic line pCYCB1;1:CYCB1;1-GUS that contains the *GUS* reporter fused to the mitotic destruction sequence (D-box) and the cyclin *CYCB1;1* promoter. In this reporter line, *GUS* is expressed upon entry into the G2 phase of cell cycle via the *CYCB1;1* promoter and its protein product is degraded upon exit from the metaphase via the D-box. Bars: 20 μm. **Figure S3.** The negative impact of *AtCHR23* cDNA over-expression on growth is enhanced by salt stress. Mean (± SD) length of the primary roots of 10-day-old wild-type (Col) and mutant seedlings grown on 75 mM NaCl. For each line 40 seedlings were measured. Asterisks indicate significant differences from the wild type: ^***^, P<0.001. **Figure S4.** Gene ontology (GO) analysis of the genes showing high variability in expression between the two replicates of *AtCHR23* over-expressing mutant grown in long-day conditions. The 298 genes showing at least 3-fold expression difference between the two replicates were classified with Classification SuperViewer. Normalised frequency of GO categories ± bootstrap SD is presented. Categories with a normalised frequency greater than 1 are over-represented and lower than 1 are under-represented. The over- or under-representation of categories highlighted in dark grey and bold are statistically significant at P<0.01; the P-value is indicated next to the SD. Click here for file

Additional file 2: Table S1 Effect of modified *AtCHR23* expression on variability of growth traits. **Table S2.** Results of nonparametric adjusted rank transform test. **Table S3.** Definition of genes tested by quantitative RT-PCR shown in Figure 10. **Table S4.** Primers used in the study. Click here for file

Additional file 3 List of potentionally DE genes in long-day mutant seedlings identified by DESeq. Click here for file

Additional file 4 Lists of genes variable between replicates of RNASeq analysis. Click here for file
