# Supplementary material for: Over-expression of Arabidopsis AtCHR23 chromatin remodeling ATPase results in increased variability of growth and gene expression
Source: BMC Plant Biol. 2014 Mar 25;14:76. doi: 10.1186/1471-2229-14-76 (PMC3987066; doi:10.1186/1471-2229-14-76)
Supplement: Additional file 1: Figure S1 — Schematic layout of T-DNA regions of plasmids used to generate transgenic Arabidopsis over-expressing AtCHR23 gene. 35S, CaMV 35S promoter; pCHR23, AtCHR23 promoter; GFP, green fluorescent protein gene; Kan, kanamycin resistance gene; BAR, barnase herbicide resistance gene; RB, LB, right and left T-DNA borders. Grey shaded box indicate the presence of 5’UTR. Figure S2. Photograph of whole-mount, GUS-stained 4-day-old roots of CYCB1;1:CYCB1;1-GUS in wild-type (left) and in AtCHR23-4ov homozygous for both transgenes. AtCHR23-4ov was crossed with the transgenic line pCYCB1;1:CYCB1;1-GUS that contains the GUS reporter fused to the mitotic destruction sequence (D-box) and the cyclin CYCB1;1 promoter. In this reporter line, GUS is expressed upon entry into the G2 phase of cell cycle via the CYCB1;1 promoter and its protein product is degraded upon exit from the metaphase via the D-box. Bars: 20 μm. Figure S3. The negative impact of AtCHR23 cDNA over-expression on growth is enhanced by salt stress. Mean (± SD) length of the primary roots of 10-day-old wild-type (Col) and mutant seedlings grown on 75 mM NaCl. For each line 40 seedlings were measured. Asterisks indicate significant differences from the wild type: ***, P<0.001. Figure S4. Gene ontology (GO) analysis of the genes showing high variability in expression between the two replicates of AtCHR23 over-expressing mutant grown in long-day conditions. The 298 genes showing at least 3-fold expression difference between the two replicates were classified with Classification SuperViewer. Normalised frequency of GO categories ± bootstrap SD is presented. Categories with a normalised frequency greater than 1 are over-represented and lower than 1 are under-represented. The over- or under-representation of categories highlighted in dark grey and bold are statistically significant at P<0.01; the P-value is indicated next to the SD. [file 1471-2229-14-76-S1.pdf]

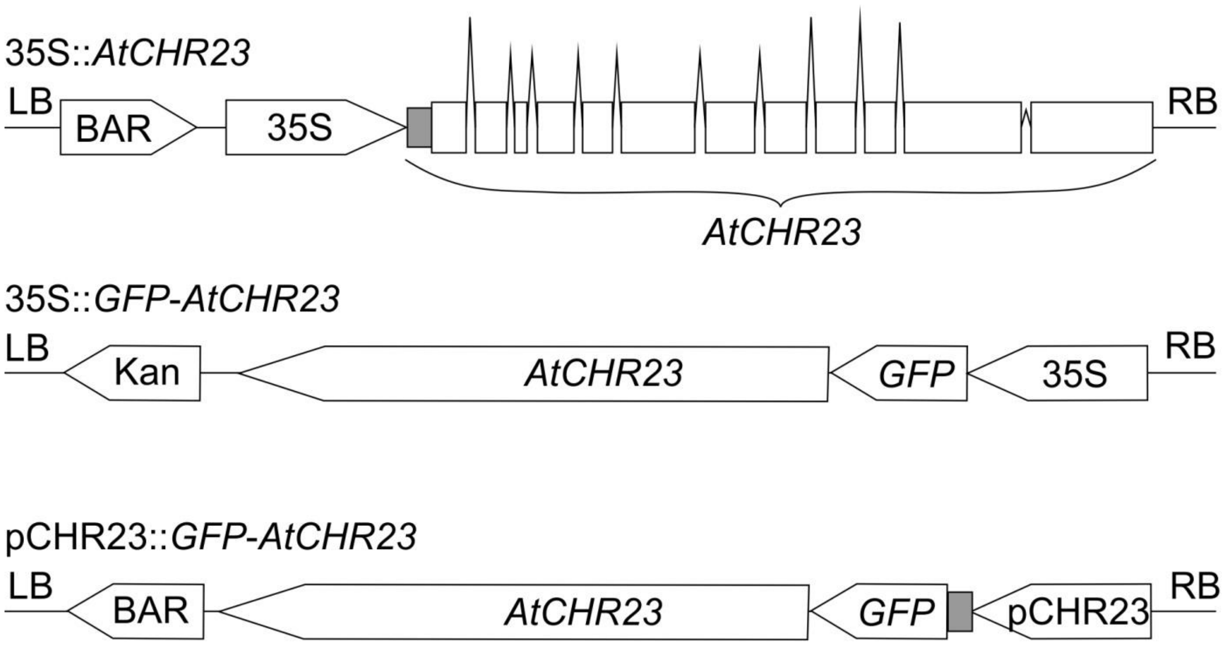

Figure S1. Schematic layout of T-DNA regions of plasmids used to generate transgenic Arabidopsis over-expressing *AtCHR23* gene. 35S, CaMV 35S promoter; pCHR23, *AtCHR23* promoter; *GFP*, green fluorescent protein gene; Kan, kanamycin resistance gene; BAR, barnase herbicide resistance gene; RB, LB, right and left T-DNA borders. Grey shaded box indicate the presence of 5’UTR.

pCYCB1;1:CYCB1;1-GUS

pCYCB1;1:CYCB1;1-GUS x *AtCHR23*-4ov

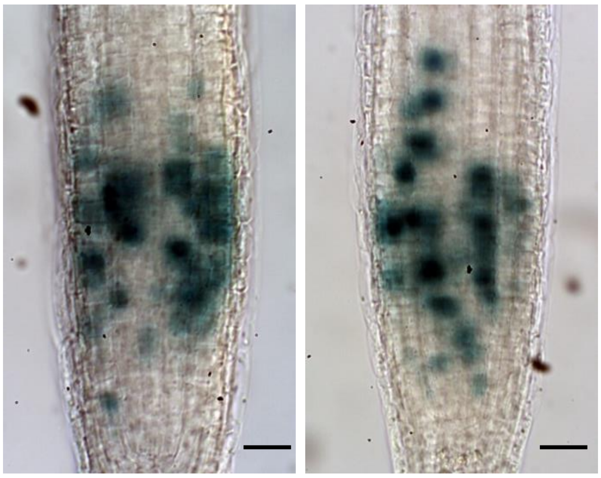

Figure S2. Photograph of whole-mount, GUS-stained 4-day-old roots of CYCB1;1:CYCB1;1-GUS in wild-type (left) and in *AtCHR23*-4ov homozygous for both transgenes. *AtCHR23*-4ov was crossed with the transgenic line pCYCB1;1:CYCB1;1-GUS that contains the *GUS* reporter fused to the mitotic destruction sequence (D-box) and the cyclin *CYCB1;1* promoter. In this reporter line, *GUS* is expressed upon entry into the G2 phase of cell cycle via the *CYCB1;1* promoter and its protein product is degraded upon exot from the metaphase via the D-box. Bars: 20 µm.

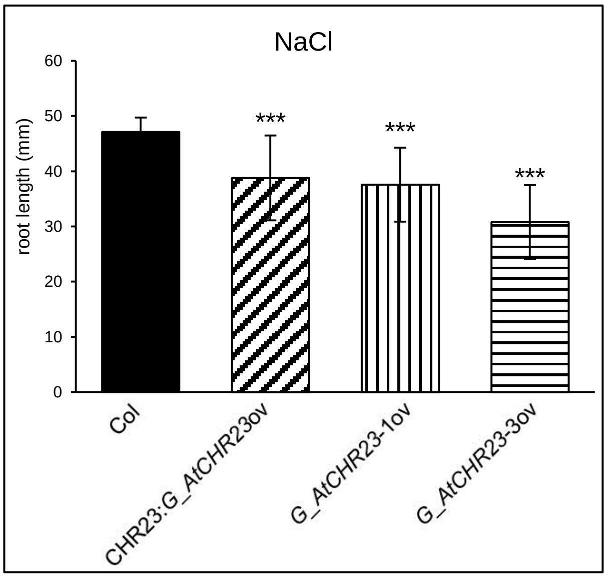

Figure S3. The negative impact of *AtCHR23* cDNA over-expression on growth is enhanced by salt stress. Mean (± SD) length of the primary roots of 10-day-old wild-type (Col) and mutant seedlings grown on 75 mM NaCl. For each line 40 seedlings were measured. Asterisks indicate significant differences from the wild type: ***, P<0.001.

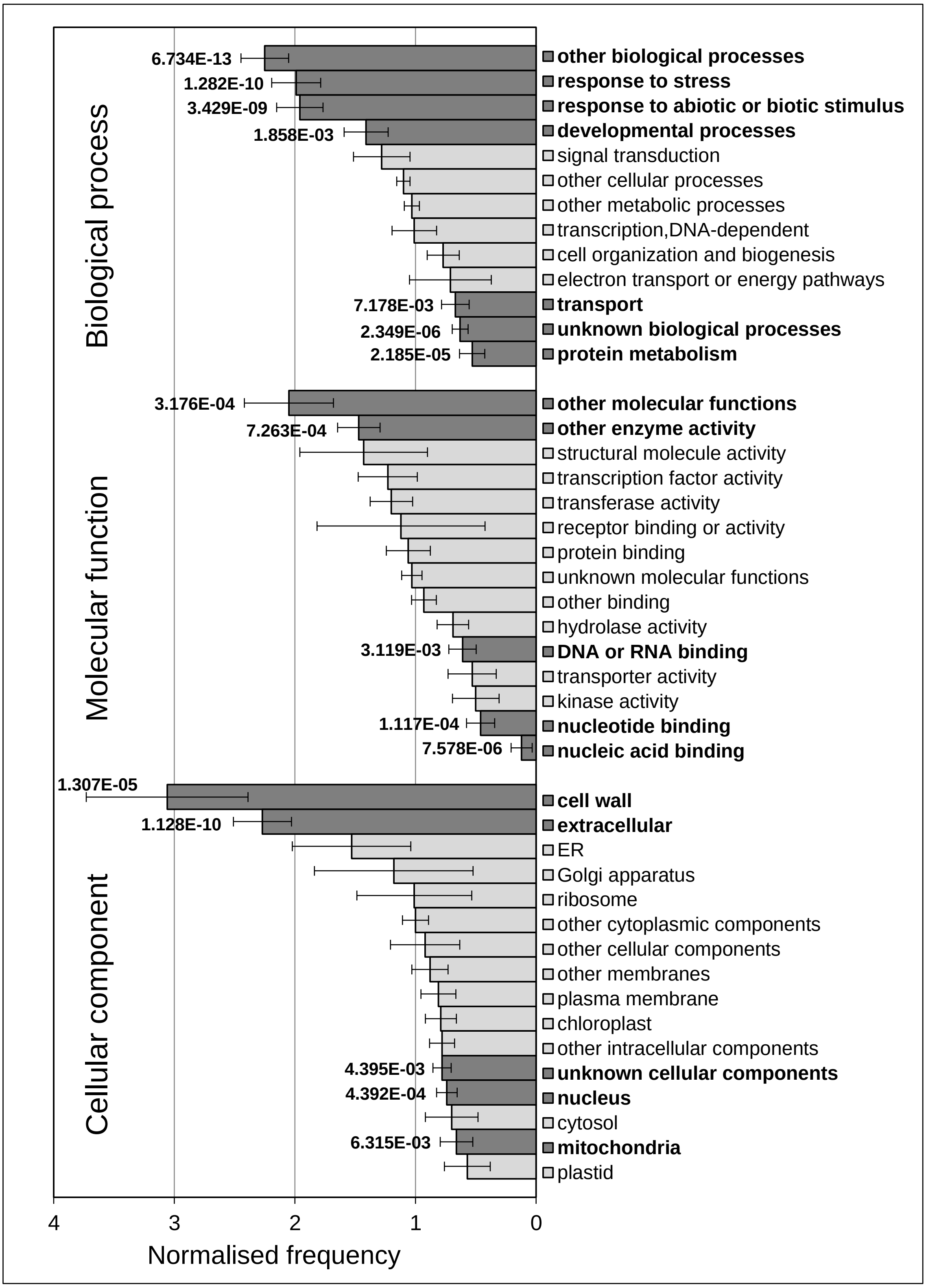

Figure S4. Gene ontology (GO) analysis of the genes showing high variability in expression between the two replicates of *AtCHR23* over-expressing mutant grown in long-day conditions. The 298 genes showing at least 3-fold expression difference between the two replicates were classified with Classification SuperViewer. Normalised frequency of GO categories ± bootstrap SD is presented. Categories with a normalised frequency greater than 1 are over-represented and lower than 1 are under-represented. The over- or under-representation of categories highlighted in dark grey and bold are statistically significant at P<0.01; the P-value is indicated next to the SD.
