# Supplementary material for: Over-expression of Arabidopsis AtCHR23 chromatin remodeling ATPase results in increased variability of growth and gene expression
Source: BMC Plant Biol. 2014 Mar 25;14:76. doi: 10.1186/1471-2229-14-76 (PMC3987066; doi:10.1186/1471-2229-14-76)
Supplement: Additional file 2: Table S1 — Effect of modified AtCHR23 expression on variability of growth traits. Table S2. Results of nonparametric adjusted rank transform test. Table S3. Definition of genes tested by quantitative RT-PCR shown in Figure 10. Table S4. Primers used in the study. [file 1471-2229-14-76-S2.docx]

**Table S1.** Effect of modified *AtCHR23* expression on growth traits.

| **Trait** | **Line** | **Mean^a^** | **CV^b^** | **VAR^c^** | **P(VAR)^d^** |
| --- | --- | --- | --- | --- | --- |
| Cotyledon area (mm^2^) | Columbia - WT | 4.675 | 0.183 | 0.730 | na |
|  | *AtCHR23*-4ov | **3.357** | 0.232 | 0.605 | ns |
|  | *AtCHR23*-5ov | **3.830** | 0.206 | 0.624 | ns |
|  | *atchr23* | 4.925 | 0.195 | 0.938 | ns |
| Hypocotyl 25°C (mm) | Columbia - WT | 0.242 | 0.117 | 7.963E-04 | na |
|  | *AtCHR23*-4ov | **0.198** | 0.159 | 9.875E-04 | ns |
|  | *AtCHR23*-5ov | **0.197** | 0.147 | 8.344E-04 | ns |
|  | *atchr23* | 0.239 | 0.132 | 9.992E-04 | ns |
| Hypocotyl 28°C (mm) | Columbia - WT | 0.933 | 0.121 | 1.269E-02 | na |
|  | *AtCHR23*-4ov | **0.754** | 0.172 | 1.686E-02 | ns |
|  | *AtCHR23*-5ov | **0.771** | 0.171 | 1.734E-02 | ns |
|  | *atchr23* | 0.926 | 0.117 | 1.171E-02 | ns |
| Root length continuous light (mm) | Columbia - WT | 38.370 | 0.118 | 20.460 | na |
|  | *AtCHR23*-4ov | **34.213** | 0.145 | 24.780 | ns |
|  | *AtCHR23*-5ov | **31.750** | 0.173 | 30.238 | ns |
|  | *atchr23* | **41.333** | 0.106 | 19.376 | ns |
| Root length: salt stress (mm) | Columbia - WT | 30.926 | 0.060 | 3.449 | na |
|  | *AtCHR23*-4ov | **21.763** | 0.160 | 12.064 | ^**^ |
|  | *AtCHR23*-5ov | **22.222** | 0.174 | 14.898 | ^***^ |
|  | *atchr23* | **31.076** | 0.058 | 3.279 | ns |
| Root length: mannitol (mm) | Columbia - WT | 32.516 | 0.087 | 7.998 | na |
|  | *AtCHR23*-4ov | **22.831** | 0.198 | 20.415 | ^**^ |
|  | *AtCHR23*-5ov | **23.871** | 0.159 | 14.453 | ns |
|  | *atchr23* | **33.953** | 0.073 | 6.135 | ns |
| Leaf area (mm^2^) | Columbia - WT | 15.732 | 0.083 | 1.718 | na |
|  | AtCHR23-4ov | **13.479** | 0.176 | 5.641 | ^**^ |
|  | AtCHR23-5ov | **14.049** | 0.176 | 6.144 | ^*^ |
|  | atchr23 | 16.506 | 0.113 | 2.462 | ns |
| Rosette diameter (cm) | Columbia - WT | 3.400 | 0.120 | 0.167 | na |
|  | AtCHR23-4ov | **2.724** | 0.165 | 0.201 | ns |
|  | AtCHR23-5ov | **3.055** | 0.190 | 0.337 | ns |
|  | atchr23 | 3.256 | 0.097 | 0.100 | ns |
| Rosette diameter: salt stress (cm) |  |  |  |  |  |
|  | Columbia - WT | 3.030 | 13.58 | 0.169 | na |
|  | AtCHR23-5ov | **2.323** | 25.77 | 0.358 | ns |
|  |  |  |  |  |  |

^a^ Mean of growth parameter indicated in left, bold indicates significant difference relative to WT as determined by Mann-Whitney U test; ^b^ coefficient of variation calculated as ratio of the standard deviation to the mean; ^c^ variance in growth parameter indicated in left; ^d^ significance of variance relative to WT as determined by Levene’s test, ns, not significant; ^*^, P<0.05; ^**^, P<0.01; ^***^; P<0.001. WT, wild-type; na, not applicable.

**Table S2.** Results of non-parametric adjusted rank transform test.

|  |  | **Salt stress** | | | **Mannitol stress** | | |
| --- | --- | --- | --- | --- | --- | --- | --- |
|  |  | **G** | **T** | **G x T** | **G** | **T** | **G x T** |
| Root length | *AtCHR23*-4ov | ^***^ | ^***^ | ^**^ | ^***^ | ^***^ | ^***^ |
|  | *AtCHR23*-5ov | ^***^ | ^***^ | ^**^ | ^***^ | ^***^ | ^**^ |
|  | *atchr23* | ^***^ | ^***^ | ^**^ | ^***^ | ^***^ | ns |
| Rosette diameter | *AtCHR23*-5ov | ^***^ | ^***^ | ns | na | na | na |

Significance of the sources of variation are Genotype (G), Treatment (T) and Genotype x Treatment interaction (G x T).

^**^, P<0.01; ^***^, P<0.001; ns, not significant; na, not applicable

**Table S3.** Definition of genes tested by quantitative RT-PCR in individual seedlings.

Names and descriptions from TAIR10 of the 14 genes tested for expression variability in 6 individual seedlings.

| **AGI number** | **Gene name** | **Function** |
| --- | --- | --- |
| *At3g11050* | FER2, FERRITIN 2 | response to oxidative stress, abscisic acid stimulus, cellular iron ion homeostasis and iron ion transport |
| *At4g31940* | CYP82C4, CYTOCHROME P450 | early Fe deficiency response |
| *At4g14690* | ELIP2, EARLY LIGHT-INDUCIBLE PROTEIN 2 | biogenesis of all chlorophyll-binding complexes |
| *At1g67105* |  | other RNA |
| *At5g19310* | ATCHR23, CHROMATIN REMODELING 23 | homeotic gene regulator |
| *At5g04220* | ATSYTC, SYNAPTOTAGMIN 3 | unknown |
| *At3g06010* | ATCHR12, CHROMATIN REMODELING 12 | temporary growth arrest in Arabidopsis upon perceiving environmental stress |
| *At3g01460* | ATMBD9, METHYL-CPG-BINDING DOMAIN 9 | modification of the FLC chromatin acetylation state |
| *At1g04220* | KCS2, 3-KETOACYL-COA SYNTHASE 2 | involved in the biosynthesis of VLCFA (very long chain fatty acids) |
| *At3g22640* | PAP85 | nutrient reservoir activity |
| *At3g12580* | ATHSP70, ARABIDOPSIS HEAT SHOCK PROTEIN 70 | ATP binding |
| *At5g02490* | ATHSP70-2, ARABIDOPSIS HEAT SHOCK PROTEIN 70-2 | protein binding |
| *At5g10140* | AGL25, AGAMOUS-LIKE 25, FLC, FLOWERING LOCUS C, FLF, FLOWERING LOCUS F, RSB6, REDUCED STEM BRANCHING 6 | MADS-box protein encoded by FLOWERING LOCUS C - transcription factor that functions as a repressor of floral transition and contributes to temperature compensation of the circadian clock |
| *At2g01422* |  | other RNA |

**Table S4.** Primers used in the study.

| **Primer name** | **Sequence 5’ > 3’** | **Used for** |
| --- | --- | --- |
| CHR23_F1 | CCCGTCTCGTTTATCTTTCG | gene cloning |
| CHR23_R1 | GGCATCTTTCTGACGCTGG | gene cloning |
| CHR23_F2 | CACCCGAGCTGAAAAACTAA | gene cloning |
| CHR23_R2 | GCTTGTATGACTTTCGCATC | gene cloning |
| CHR23_F3 | GATCGTGCTCATCGGATAG | gene cloning |
| CHR23_R3 | TCAGTTTCGTTTACTTCCTTTT | gene cloning |
| CHR23_F4 | GGGGCAACTTTGTACAAAAAAGTTG  GCATGGTGAAGCAGCTACAAG | gene cloning |
| CHR23_R4 | GGGGACCACTTTGTACAAGAAAGCT  GGGTCTCAGTTTCGTTTACTTCCTTTTGAG | gene cloning |
| pCHR23_F | CACCGCTTCGATAAAAAGAGTCAAAG | promoter cloning |
| pCHR23_R | GGCGGGAGTTTCTAATTAGA | promoter cloning |
| qCHR23_F | CTAGGAACTGGCTACCGGA | qRT-PCR |
| qCHR23_R | AGCGACCATAGTTCTTGCAGA | qRT-PCR |
| At1g67105_F | CATCTTCGTCACCTCCGATT | qRT-PCR |
| At1g67105_R | TCAGTGCGATGGGTAGACTG | qRT-PCR |
| At3g01460_F | ATGGTTTCCCTGAGCAAAAGGGTAG | qRT-PCR |
| At3g01460_R | ACTGCATCGGACATCCATTCTTAGC | qRT-PCR |
| At3g06010_F | TTCCACTGCACAAGACAGAAG | qRT-PCR |
| At3g06010_R | TCTTGCTCTTGCATCAGACG | qRT-PCR |
| At3g11050_F | TCGAACCTTTTGAGGAGGTG | qRT-PCR |
| At3g11050_R | AATCGTCGGAGAACTTGTGG | qRT-PCR |
| At4g14690_F | CGCCATGGAGTTATCAAAGG | qRT-PCR |
| At4g14690_R | CCTTTTGACTTTGCCTCTGC | qRT-PCR |
| At4g31940_F | GCAACCATCGAGCTTCTTTC | qRT-PCR |
| At4g31940_R | CTGGTTTTGTACCGCCATTC | qRT-PCR |
| At5g04220_F | AGATGTCGAGGGCAAGAAGA | qRT-PCR |
| At5g04220_R | GAAAGTGAAAGCCGGTCCCT | qRT-PCR |
